# The Annual Meeting of the Erie County Medical Society

**Published:** 1854-02

**Authors:** 


*The Annual Meeting of the Erie County Medical Society.*—The annual meeting of the Medical Society of the County of Erie, convened in this city on Tuesday the 10th of January, at 10½ o'clock, A. M., the president, Dr. Strong, presiding.

Forty members were present during the morning and participated in the business of the day.

Doctors S. B. Hunt, C. L. Dayton, T. W. Wood, and T. F. Rochester were admitted to membership with the society.

The usual reports from the librarian and treasurer were made.

A committee appointed at the last annual meeting, to purchase books, made a report, recommending the purchase, for the society library, of the "Sydenham Society's Publications," and the volumes of Transactions of the American Medical Association, to complete the set.

The recommendations of the committee were adopted, the committee continued for the ensuing year, and the following resolution, proposed by Dr. White, unanimously adopted:

*Resolved,*—That all unexpended funds, left after paying all contingent expenses, be placed at the disposal of the committee on purchase of books for insurance, repairs, and purchase of additions to the library.

The following resolution was also offered by Dr. White:

*Resolved,*—That a committee of three be appointed to report a tariff of prices for medical services and appointments in and for the county of Erie, and city of Buffalo, below which it shall be dishonorable to do the business, or to accept the appointments.

The injustice suffered by the profession at the hands of the public authorities, and the unprofessional character, and the injurious effects resulting from physicians entering into a pecuniary competition, or *bidding* for appointments and business, were urged by the mover and seconded by many others. The resolution was unanimously adopted, and a committee appointed, consisting of Drs. Samo, House and Wyckoff.

Dr. Hunt was appointed orator for the semi-annual meeting.

A letter was read from Dr. Mackay, the orator for the day, stating his inability to perform the duty at this time, but intimating his willingness to do so at any future time. An invitation was extended to him to address the society at its June session.

Several appropriations were voted to meet current expenses.

The annual choice of officers were made, and the following gentlemen elected to the respective offices for the ensuing year:

| | | |
|---|---|---|
| Dr. J. G. House, | . . . . | *President,* |
| " J. P. White, | . . . . . | *Vice-President.* |
| " J. M. Newman, | . . . . . | *Secretary.* |
| " S. G. Bailey, | . . . . . | *Treasurer.* |
| " J. B. Samo, | . . . . . | *Librarian.* |

*Primary Board.*—Drs. S. Eastman, W. Ring, J. S. Hawley.

| | | |
|---|---|---|
| *Censors.*—Dr. F. H. Hamilton, | *Exam. in* | *Anat. Phys. and Surg.* |
| " J. B. Samo, | " | *Pract. Med. and Obstet.* |
| " W. Treat, | " | *Chemis. and Pharma.* |
| " W. Van Pelt, | " | *Mat. Med. and Botany.* |
| " H. M. Congar, | " | *Med. Juris. and Pathol.* |

*Delegates to the State Medical Society.*—Drs. Congar, Rochester, White and House.

The *delegates* to the *American Medical Association* to be appointed by the president and secretary.

In consequence of the cold and uncomfortable state of the hall, the president's valedictory address was, on motion, postponed until after dinner, the committee having that *exercise* in charge, reporting that arrangements had been made for the entertainment of the members at the American Hotel.

At three o'clock the society convened in one of the parlors of the American, and was called to order by the president.

The following resolution was presented by Dr. Hunt:

*Resolved,*—That this society do earnestly urge upon the legislature of the state of New York, the passage of a bill legalizing the dissection of the human body, and making sufficient provision for the supply of convict and pauper bodies unclaimed by friends, to medical men applying for them.

The importance of this subject to the profession was discussed, and the resolution unanimously adopted.

A *move* was then made for the dinner table, and thirty gentlemen sat down to an admirable repast, spread in the best style of Mr. Hodge, the worthy host of the American. Ample time being allowed for the discussion of the good things set before them, and replenishing the wants of the physical man, the cloth was removed and the president read his valedictory address.

It was a paper admirably adapted to the occasion. It presented first, a rapid review of the principal events in the society's history for the past year, and then passed on to many a wholesome suggestion for improvement in medical knowledge, and the amenities of professional intercourse. The whole was replete with sound sense, and was favorably received by the society.

The balance of the session was pleasantly spent in the interchange of expressions of mutual good feeling, and of those generous sentiments engendered by such a festive occasion, and with toasts and speeches the members whiled away the hours until 8 o'clock, at which time, after having made arrangements for a dinner at the semi-annual meeting, the society *adjourned.*

